# Multi-omic single cell analysis resolves novel stromal cell populations in healthy and diseased human tendon

**DOI:** 10.1038/s41598-020-70786-5

**Published:** 2020-09-03

**Authors:** Adrian R. Kendal, Thomas Layton, Hussein Al-Mossawi, Louise Appleton, Stephanie Dakin, Rick Brown, Constantinos Loizou, Mark Rogers, Robert Sharp, Andrew Carr

**Affiliations:** 1 The Nuffield Department of Orthopaedics, Rheumatology and Musculoskeletal Sciences, Botnar Research Centre, Windmill Road, Oxford, OX3 7LD UK; 2 Nuffield Orthopaedic Centre, Windmill Road, Oxford, OX3 7LD UK

**Keywords:** Gene expression analysis, Ageing, RNA sequencing, Proteomics, Inflammation, Chronic inflammation

## Abstract

Tendinopathy accounts for over 30% of primary care consultations and represents a growing healthcare challenge in an active and increasingly ageing population. Recognising critical cells involved in tendinopathy is essential in developing therapeutics to meet this challenge. Tendon cells are heterogenous and sparsely distributed in a dense collagen matrix; limiting previous methods to investigate cell characteristics ex vivo. We applied next generation CITE-sequencing; combining surface proteomics with in-depth, unbiased gene expression analysis of > 6400 single cells ex vivo from 11 chronically tendinopathic and 8 healthy human tendons. Immunohistochemistry validated the single cell findings. For the first time we show that human tendon harbours at least five distinct *COL1A1/2* expressing tenocyte populations in addition to endothelial cells, T-cells, and monocytes. These consist of *KRT7/SCX*+ cells expressing microfibril associated genes, *PTX3*+ cells co-expressing high levels of pro-inflammatory markers, *APOD*+ fibro–adipogenic progenitors, *TPPP3/PRG4*+ chondrogenic cells, and *ITGA7*+ smooth muscle-mesenchymal cells. Surface proteomic analysis identified markers by which these sub-classes could be isolated and targeted in future. Chronic tendinopathy was associated with increased expression of pro-inflammatory markers *PTX3*, *CXCL1, CXCL6, CXCL8,* and *PDPN* by microfibril associated tenocytes. Diseased endothelium had increased expression of chemokine and alarmin genes including *IL33.*

## Introduction

Musculoskeletal disorders are responsible for the second largest number of years lived with disability worldwide^1^. The morbidity associated with tendon degeneration in an ageing, and active, population represents an escalating challenge to healthcare services. It affects up to third of the population, accounting for 30% of primary care consultations^2–4^. Tendon disorders commonly affect the lower limb and result in long term pain and disability. These range from isolated tendon rupture (most commonly the Achilles tendon), to disease that drives complex foot deformity, such as adult acquired flat foot deformity (AAFD) affecting 2–3% of the adult population^5–7^.

Early research into patients with Marfan syndrome demonstrated that tendon cells are surrounded by peri-cellular matrix microfibrils formed by fibrillin chains and bound ancillary proteins (including versican, fibulin, matrix associated glycoproteins). They represent a mechanism for altering growth factor signalling (e.g. sequestering TGFβ), controlling morphogenic gradients, and influencing cell interactions with the extracellular collagen matrix^8–10^. They may allow resident tenocytes to sample, respond and influence tendon structure and function^11^. The development of new therapies requires an understanding of how key subpopulations of tendon cells/fibroblasts are able to maintain a dense tendon extracellular matrix and importantly, how they survey and respond to events which threaten tissue homeostasis.

The bulk of healthy tendon consists of dense collagen matrix and residing cells tend to be sparse, heterogenous and autofluorescent; so limiting previous techniques of studying cells of interest, such as flow cytometry. Moreover the mechano-sensitivity of tendon fibroblasts risks confounding their interrogation in vitro including gene expression analysis^12^. It is currently not possible to define, isolate and target specific subpopulations of matrix producing cells involved in human tendon disease. Single cell RNA sequencing offers an unbiased and sensitive inventory of the transcriptome of individual cells and allows characterisation of cell subtypes based on shared and differential gene expression data^13^. This approach has been successfully used to characterise cell types is mouse tissue^14–17^.

CITE-Seq (Cellular Indexing of Transcriptomes and Epitopes by Sequencing) is a novel iteration of single cell sequencing that uses oligonucleotide barcodes conjugated to monoclonal antibodies to combine surface proteomics with single cell RNA/transcriptomic information. To our knowledge this is the first time CITE-Seq has been applied to healthy and diseased human tendon. We have identified multiple subpopulations of cells in human tendon, five of which show increased expression of *COL1A1/2* genes. These include two groups that co-express microfibril genes, a group expressing genes associated with fibro–adipogenic progenitors (FAPs), a *TPPP3/PRG4*+ chondrogenic group and *ITGA7*+ smooth muscle-mesenchymal cells (SMMCs), previously described in mouse but not in human tendon^16,17^. These findings support the presence of multiple specialised tendon cell subtypes and open new avenues to interrogate key cell pathways that underpin chronic tendon disease.

## Results

### A single cell atlas of human tendon in health and disease

In order to characterise healthy and diseased human tendon cell subtypes, we performed scRNA-seq and CITE-seq integrated analysis of cells from eight healthy and eleven diseased tendon samples (Table 1). Cells were incubated with one of eight surface hashing antibodies so that their sample of origin could be identified following sequencing across three lanes (Supplemental Table 1). After normalization and quality control of the transcriptome data, cells were selected based on high ‘expression level’ of their donor specific surface hashing antibody and low ‘expression level’ of the remaining hashing antibodies (Supplemental Figure 1).

**Table 1:** Patient donor demographics of healthy and disease tendon.

OMB id	Age (years)	Sex	Tendon	Clinical disease
**Healthy**				
0963	24	F	Hamstring	ACL rupture
0924*	48	F	Hamstring	ACL rupture
0938	49	F	Hamstring	ACL rupture
0925*	21	M	Tibialis posterior	Tendon transfer for foot drop
0937*	27	M	Hamstring	ACL rupture
1105	34	M	Flexor hallucis longus	Tendon transfer for missed Achilles rupture
0954	43	M	Hamstring	ACL rupture
0988h*	68	M	Flexor hallucis longus	Tendon transfer for missed Achilles rupture
**Diseased**				
0933	23	F	Diseased Achilles	Chronic Achilles tendinopathy. Intractable pain. Previous Haglund’s deformity excision
0932*	52	F	2nd toe extensor	Fixed hammer toe PIPJ deformity, arthrosis
0951*	55	F	Diseased Achilles	Chronic Achilles tendinopathy. Intractable pain. Haglunds deformity
0957a	60	F	2nd toe extensor	Fixed hammer toe PIPJ deformity, arthrosis
0957b	60	F	3rd toe extensor	Fixed hammer toe PIPJ deformity, arthrosis
0949	67	F	2nd toe extensor	Fixed hammer toe PIPJ deformity, arthrosis
0931*	68	F	2nd toe extensor	Fixed hammer toe PIPJ deformity, arthrosis
0964	34	M	Diseased peroneus long	Revision right peroneal brevis debridement for intractable pain
0992	53	M	2nd toe extensor	Fixed hammer toe PIPJ deformity, arthrosis
0988p*	68	M	Diseased Achilles	Chronic Achilles tendinopathy. Intractable pain
0989*	70	M	Diseased peroneus long	Chronic peroneal tendinopathy. Intractable pain

Healthy tendon was obtained from patients who underwent tendon transfer procedures for reconstruction of knee anterior cruciate ligament (ACL), or ruptured Achilles tendon or to treat foot drop. Diseased tendon samples were restricted to patients who had chronic tendinopathy and medically intractable pain. Diseased tendon samples were from significantly older patients than healthy tendon (mean age 55 years vs. 39 years, *p* = 0.04).

*Used for immuno-histochemistry.

**Figure 1:**
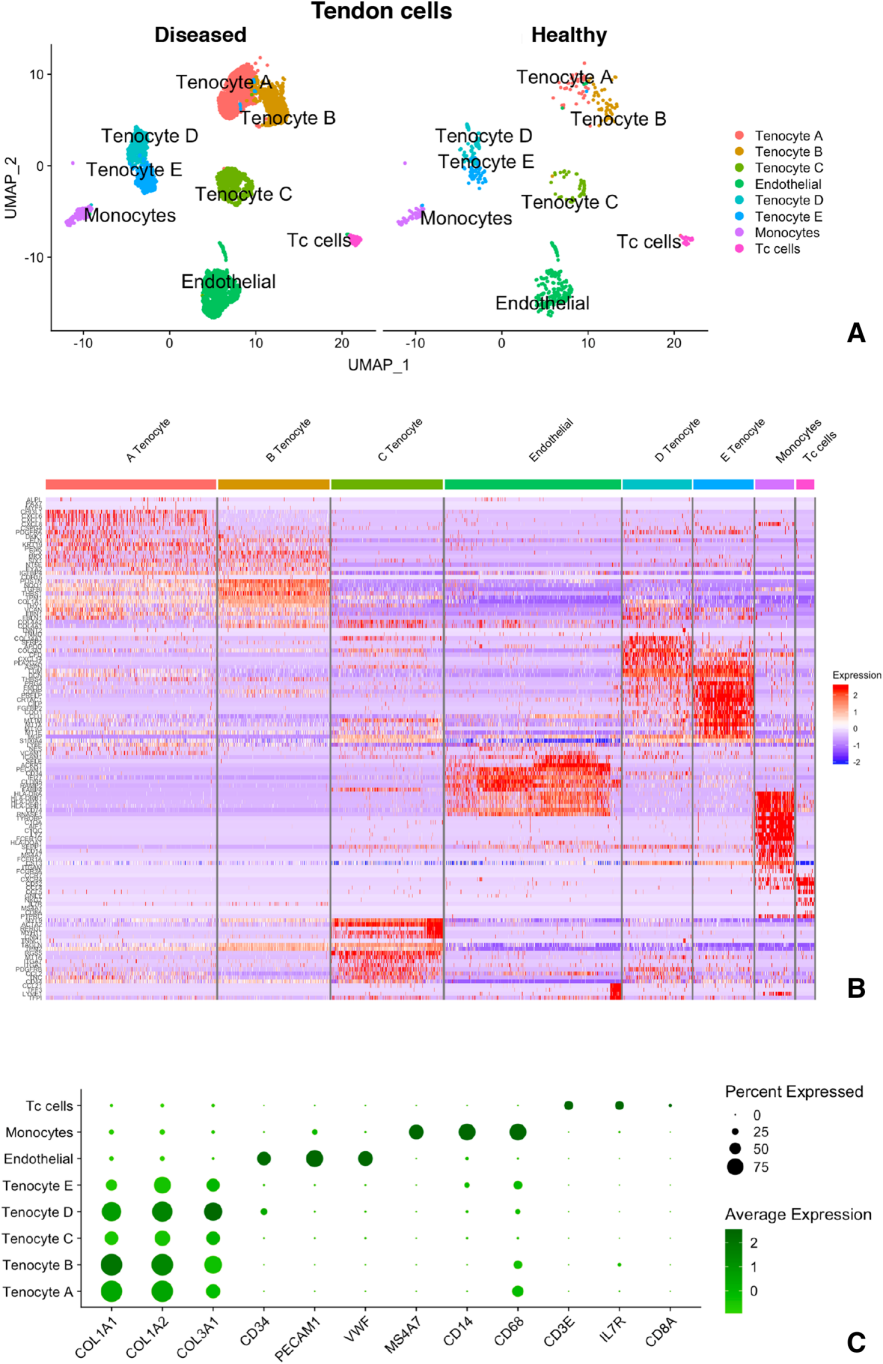
A single cell gene atlas of human tendon in health and disease. (**A**) Ex vivo single cell transcriptomic Uniform Manifold Approximation and Projection (UMAP) dimensionality reduction revealed eight distinct cell populations on clustering based on unbiased differential gene expression of the integrated data set. Each cluster is composed of cells originating from both healthy and diseased samples. (**B**) RNA expression heatmap for clusters (coloured columns) and genes (rows) of the total data set. Genes were chosen based on unbiased analysis of the top 50 differentially expressed genes, the top 4 genes per cluster and literature selected markers. Blue indicates a relative decrease in expression of a particular gene, while red indicates increased expression of a gene for each cell. (**C**) Dot plot summarising the expression pattern of selected markers to identify each major cluster of the total data set. The percentage of cells (size of dot) and average expression level (intensity of colour) are shown for each gene.

Data was derived, post quality control, for 6,433 cells obtained immediately ex-vivo from digested healthy and diseased tendon. Unsupervised graph based clustering was performed on the integrated data set and UMAP (uniform manifold approximation and projection^18^) resolved eight distinct transcriptomic clusters found in both healthy and diseased tendon (Fig. 1A). Consistent with previous observations, a greater number of cells were obtained from diseased compared to healthy tendon (Fig. 1A and Supplemental Table 2). Each cluster was further annotated by combining the top differentially expressed genes with a set of literature-defined gene markers and revealed five *COL1A1/2* expressing tenocyte clusters (initially labelled ‘Tenocyte A–E’), monocytes, Tc lymphocytes and a group of combined endothelial cells (Fig. 1B). The dot plot in Fig. 1C summarises the average expression level of a triad of genes used to help grossly distinguish Tenocyte A–E clusters, Endothelial, Monocytes and Tc cell clusters.

### Multiple distinct tenocyte populations reside in human tendon

**Figure 2:**
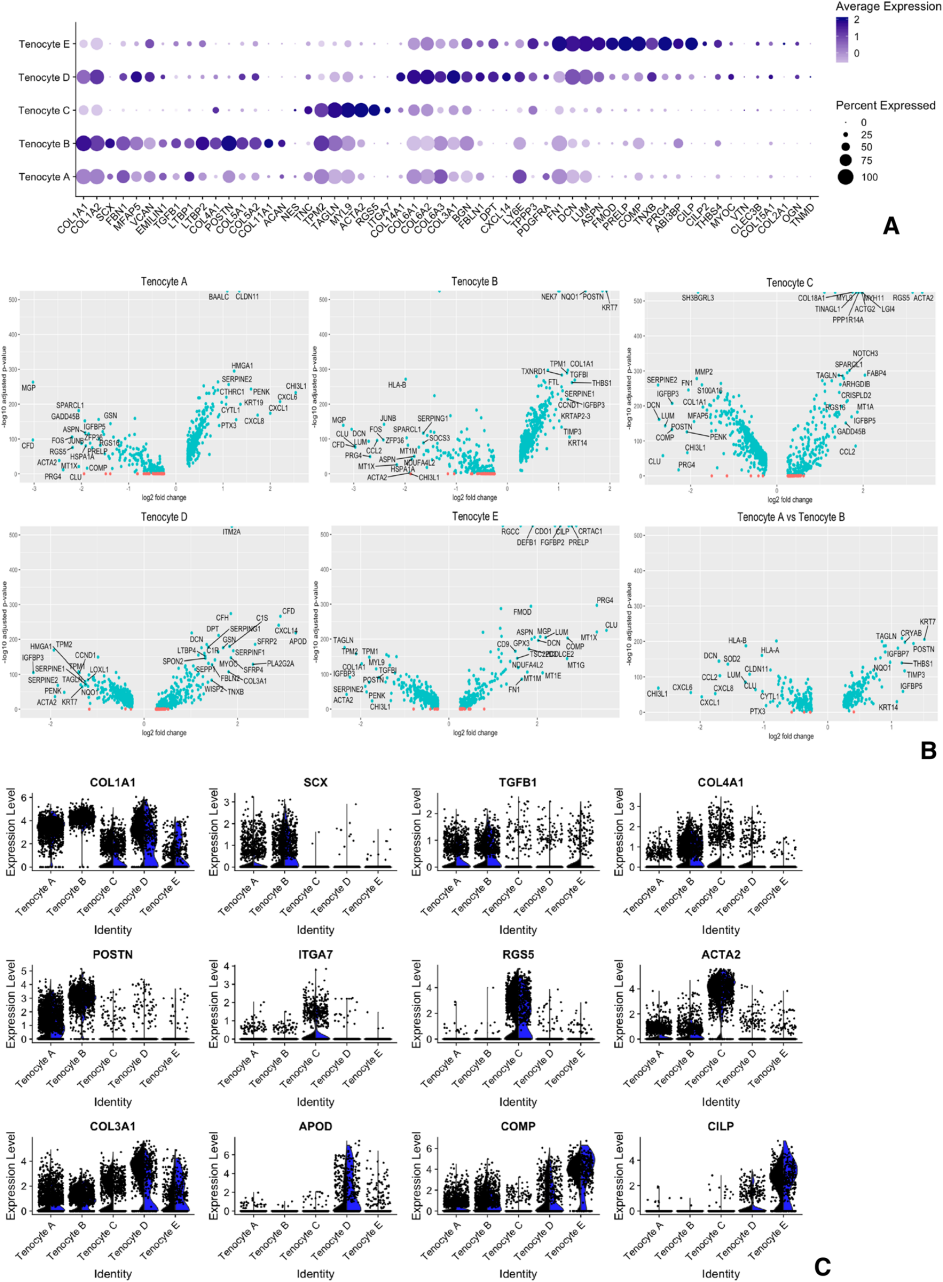
Analysis of five populations of *COL1A1/2* expressing tenocytes in healthy and diseased tendon. (**A**) Split dot plot of clusters expressing high levels of *COL1A1/2*. The percentage of cells (size of dot) and the cluster’s average expression level (intensity of colour) of genes coding for tendon matrix were compared across the five tenocyte populations for the combined data set. (**B**) Volcano plots showing differential average gene expression of each Tenocyte A-E cluster compared to the remaining clusters. Blue dots indicate adjusted *p* value < 0.05 and named genes indicate log2 fold change of > 1. (**C**) Split Violin plots of selected matrix genes for diseased (black) versus healthy (blue) tendon cells of *COL1A1/2* expressing clusters. Each dot represents the gene expression level of an individual cell.

The five cell clusters that expressed tendon matrix *COL1A1/2* were provisionally labelled Tenocyte A–E. These were generated based on an unbiased analysis of differential gene expression across the integrated data set and as such could be an artefact of random gene expression with little relevance to tendon cells. In order to test this, the expression of genes coding for the most common matrix proteins found in human tendon was analysed across the Tenocyte A–E clusters. A comprehensive extracellular proteome has previously been described for healthy, diseased and ageing human tendon and served as a reference catalogue^19^. Those genes coding for the commonest matrix proteins were applied to our data set. The differential expression of these fifty-six pre-determined genes mapped onto the *COL1A1/2*+ clusters such that the same five Tenocyte A–E clusters could still be discerned and groups of up-regulated matrix genes further delineated each cluster (Fig. 2A, dot plot).

To study further transcriptomic differences between the clusters, the average expression level of genes in a given Tenocyte cluster was directly compared to the remaining four Tenocyte clusters (Fig. 2B, volcano plots). Together these demonstrated that Tenocyte A and Tenocyte B clusters contained cells expressing *SCX* as well as genes associated with extracellular tendon microfibrils (*FBN1, MFAP5, VCAN, EMILIN1*), and TGFβ signalling (e.g. *TGFB1*, *LTBP1*, *LTBP2*). In order to discriminated between these two clusters, their gene expression profiles were compared directly to each other (Fig. 2B, final volcano plot). Tenocyte A cells displayed up-regulation of *KRT19, CHI3L, CLDN11, BAALC, PENK, SERPINE2* and pro-inflammatory genes *CXCL1, CXCL6, CXCL8, PTX3*. In comparison, Tenocyte B cells expressed higher levels of *COL4A1, KRT7, POSTN, TAGLN, THBS1, TIMP3, IGFBP5/7* and *LTBP2*.

Tenocyte D and Tenocyte E clusters shared features of fibro–adipogenic progenitors (FAPs) including up-regulation of *COL3A1, GSN, LUM, DCN, LY6E, PDGFRA* and *CXCL14* (Fig. 2). Cells in the Tenocyte D cluster in particular showed up-regulation of *COL6A1/2/3, BGN, FBLN2, APOD, PLA2G2A* and *CXCL14*. Whereas, Tenocyte E cells were found to have increased expression of *PRG4*, *TPPP3*, *DCN, CLU, LUM, PRELP, PCOLCE2, COMP, FMOD, CRTAC1* and *CILP1/2*.

The five Tenocyte A–E clusters were observed across healthy, diseased, male and female tendon as well as across the different types (anatomical locations) of tendons sampled (Supplemental Figure 3). The percentage of cells forming Tenocyte A–E clusters varied for each of the donor tendon types and in male versus female tendon. The proportion of Tenocytes A–E was similar in hamstring tendon, whereas a larger proportion of female, diseased and digital tendons (flexor hallucis longus, toe extensors) were Tenocytes A/B and a larger proportion of male foot invertors/evertors (peroneal and tibialis posterior tendon) were Tenocyte E (Supplemental Figure 3B,C).

### Single cell surface proteomics reveals a perivascular niche in human tendon

The gene expression signature of cells in the Tenocyte C cluster were characteristic of *ITGA7*+ cells recently described in mouse muscle and named smooth muscle-mesenchymal cells (SMMCs)^16^. These cells have a low expression level of *VCAM1*, and increased expression of *TAGLN, MYL9, ACTA2, RGS5* and *ITGA7* (Fig. 2A,C). There was an associated high expression level of basement membrane *COL4A1* (Figs. 1B, 2A).

**Figure 3:**
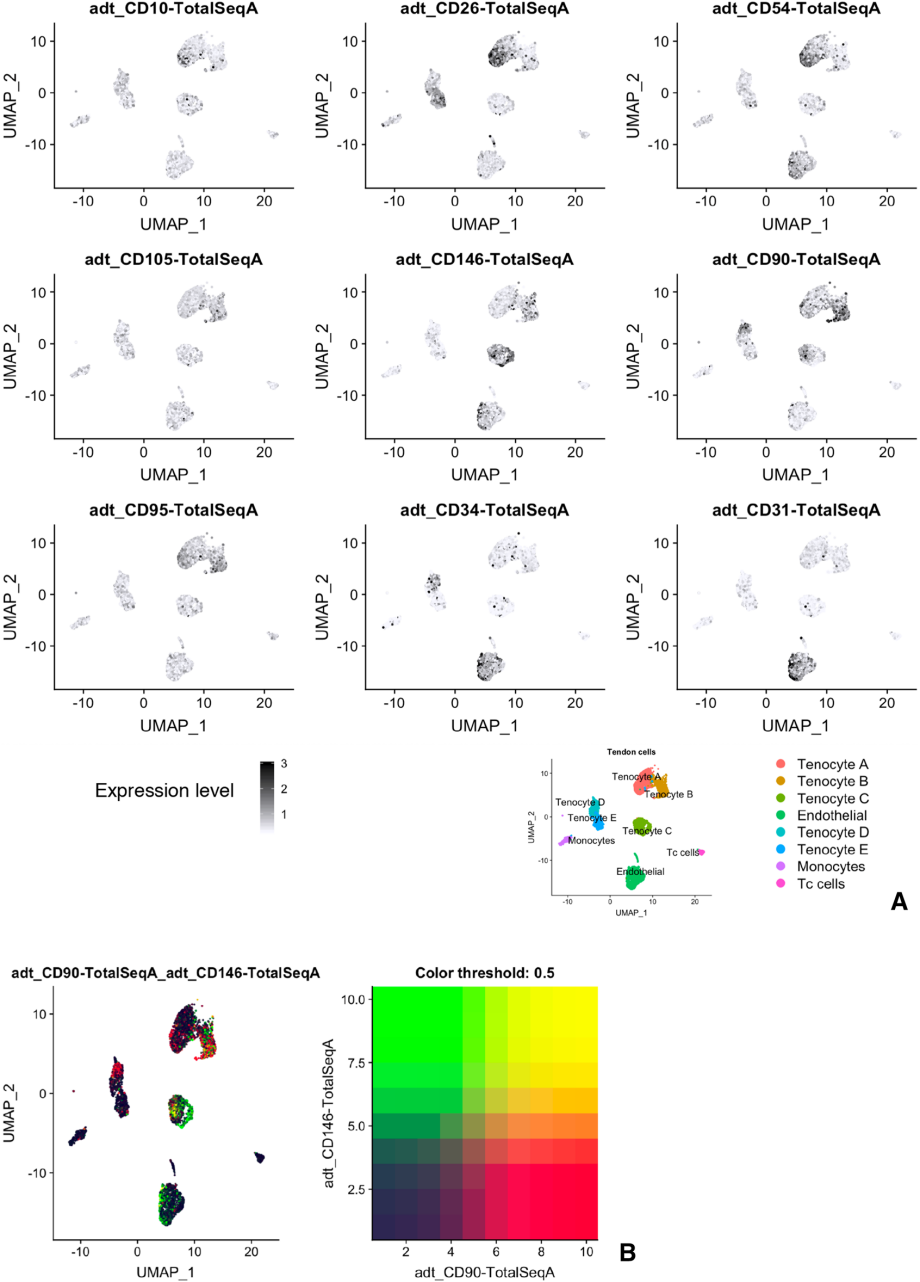
Validation of distinct clusters in human tendon using surface protein quantification. (**A**) Feature plot of ex vivo cells combined from healthy and diseased tendon incubated with oligonucleotide barcoded antibodies that recognise surface proteins. (**B**) Combined feature plot demonstrating high co-expression (yellow) of surface CD90 (red) and CD146 (green) on cells in SMMCs of Tenocyte C.

**Figure 4:**
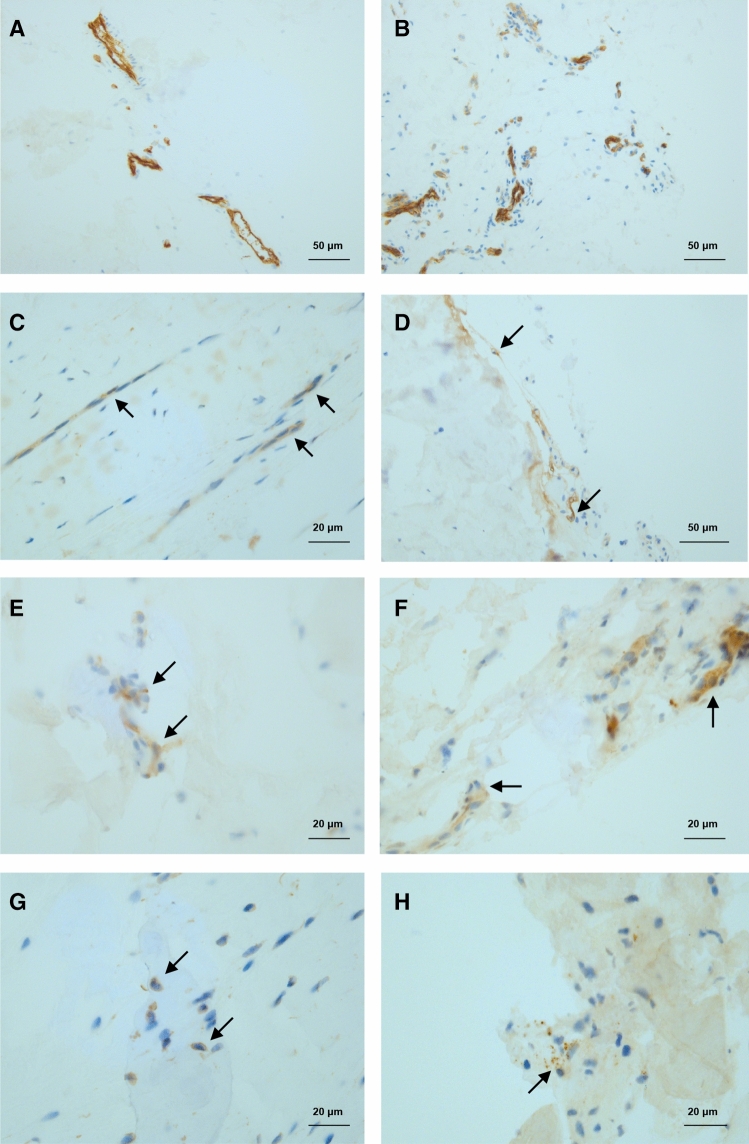
Immuno-histochemistry of human tendon demonstrating resident cell subtypes. The images are representative of five healthy and five diseased human tendon samples. Endothelial cells of healthy tendon stained with (**A**) anti-SMA and (**B**) anti-CD31 mAb. (**C**) Anti-PTX3 mAb staining of healthy tendon (putative Tenocyte A). (**D**) Anti-Periostin mAb staining of healthy tendon (putative Tenocyte B). (**E**) Anti-Cytokeratin 7 mAb of diseased tendon (putative Tenocyte B). (**F**) Anti-ITGA7 mAb of healthy tendon (putative Tenocyte C). (**G**) Anti-CXCL14 mAb staining of diseased tendon (putative Tenocyte D). (**H**) Intracellular staining for Matrix-gla protein (MGP) of healthy tendon (putative Tenocyte E).

Figure 3 shows the CITE-Seq proteomic analysis of the integrated disease and healthy tendon data set using oligonucleotide conjugated monoclonal antibodies to recognise surface proteins. SMMCs (Tenocyte C cluster) were found to co-express high levels of surface CD90 and CD146 proteins (Fig. 3 and Table 2). In addition, immunohistochemistry demonstrated that ITGA7 positive staining cells were found in human tissue, clustered around vessels (Fig. 4).

**Table 2:** Table summary of cell surface marker expression of non immune cell clusters.

Cluster	CD10	CD26	CD54	CD105	CD146	CD90	CD34	CD31
Tenocyte A (PTX3+)	**+**	**+**	**+**	Low	**−**	Low	**−**	**−**
Tenocyte B (SCX+)	**−**	**−**	Low	**+**	**+**	**+**	**−**	**−**
Tenocyte C (SMMC)	**−**	**−**	Low	Low	**+**	**+**	**−**	**−**
Tenocyte D (FAP)	**−**	**−**	Low	Low	**−**	**+**	**+**	**−**
Tenocyte E (PRG4+)	**−**	**+**	Low	Low	**−**	Low	**−**	**−**
Endothelial	**−**	**−**	**+**	Low	**+**	Low	**+**	**+**

Summary of surface markers based on expression levels for oligonucleotide barcoded antibodies with reference to Fig. 3A. ‘**+**’ indicates expression level of at least 2 × baseline (black in feature plot of Fig. 3A), ‘low’ indicates expression level > 1 and < 2 (grey in feature plot of Fig. 3A) and ‘−’ indicates expression level < 1 (off white in feature plot of Fig. 3A).

Single cell surface proteomics further distinguished endothelial cells (CD34^+^CD31^+^) from Tenocyte A (CD10^+^CD26^+^CD54^+^/CD90^low^CD95^low^), Tenocyte B cells (CD90^+^CD105^+^CD146^+^), Tenocyte D cells (CD34^+^CD90^+^/CD146^low^CD105^low^) and Tenocyte E cells (CD26^+^/CD90^low^CD54^low^CD10^neg^).

### Discrete cell subtypes are demonstrated in situ in human tissue

Immuno-histochemistry was used to investigate whether the five Tenocyte A–E clusters generated by single cell transcriptomic analysis could be found within human tendon. Genes demonstrating the greatest increase in relative expression (log2 fold change) for each Tenocyte cluster were found by comparing the average expression in that cluster against the remaining Tenocyte clusters (Fig. 2B and Supplemental Table 3). These genes included *PTX3* (Pentraxin 3)*, POSTN* (Periostin), *KRT7* (Cytokeratin 7), *ITGA7* (Integrin Subunit Alpha 7), *CXCL14*, and *MGP* (Matrix gla protein).

Five healthy and five diseased tendon samples were formalin fixed and stained with anti-Pentraxin 3 (PTX3) for Tenocyte A, anti-Periostin and anti-Cytokeratin 7 for Tenocyte B, anti-Integrin Subunit Alpha 7 (ITGA7) for Tenocyte C, anti-CXCL14 for Tenocyte D, and anti-Matrix gla protein (MGP) antibodies for Tenocyte E.

SMA+ and CD31+ endothelial cells were observed surrounding blood vessels (Fig. 4A,B). Pentraxin 3 (PTX3) staining demonstrated long linear chains of tendon cells surrounded by matrix (Fig. 4C). Groups of Cytokeratin 7 and Periostin stained cells were found near the periphery of tendon samples or were clustered within the main substance (Fig. 4D,E). ITGA7 stained cells tended to be situated near blood vessels in small clusters and occasionally formed shorter strings of cells (Fig. 4F). Some of these formed chains of cells but without the distinct morphology of PTX3 cells. A minority of cells stained positively for the secreted chemokine CXCL14 and a small group were positive for the intracellular Matrix gla protein (Fig. 4G,H).

### Stromal cell populations are dynamic in human tendon disease.

**Figure 5:**
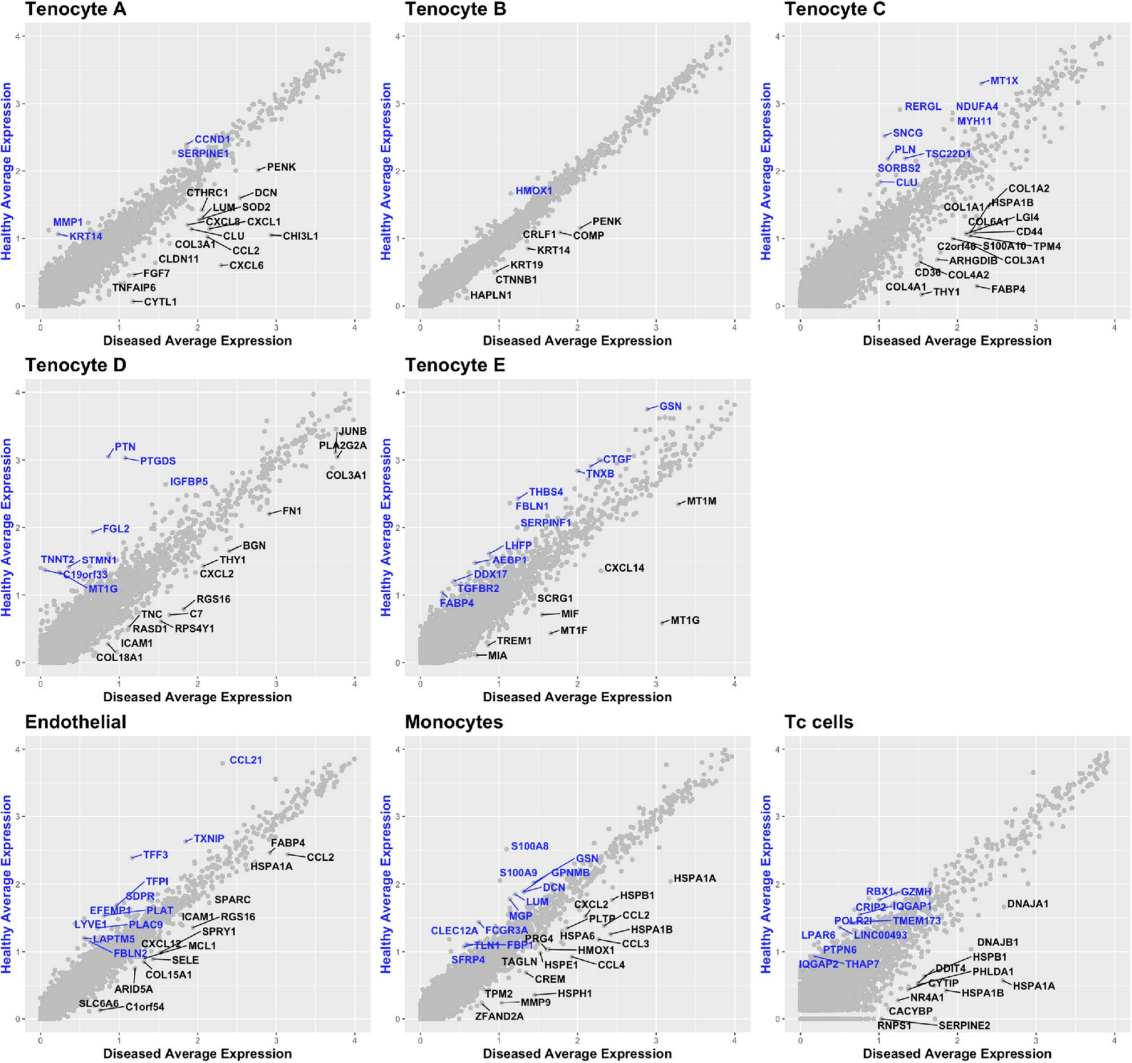
Differential gene expression of diseased versus healthy tendon cell populations. Scatter plots of average gene expression of cells in each cluster. Those genes exhibiting increased expression in cells derived from healthy compared to diseased tendon are highlighted in blue. Genes exhibiting increased expression in cells derived from diseased compared to healthy tendon are highlighted in black for each cluster.

The average expression of genes in healthy versus diseased cells was compared for each of the main cell clusters (Fig. 5 and Supplemental Table 3). Diseased cells across multiple Tenocyte clusters were found to have increased expression of matrix associated genes. Diseased Tenocyte C cells up-regulated COL1A1/2, COL3A1, COL4A1/2, COL6A1. Diseased Tenocyte D cells showed increased expression of COL3A1 compared to healthy cells. COL3A1, DCN, and LUM were increased in diseased Tenocyte A cells.

**Figure 6:**
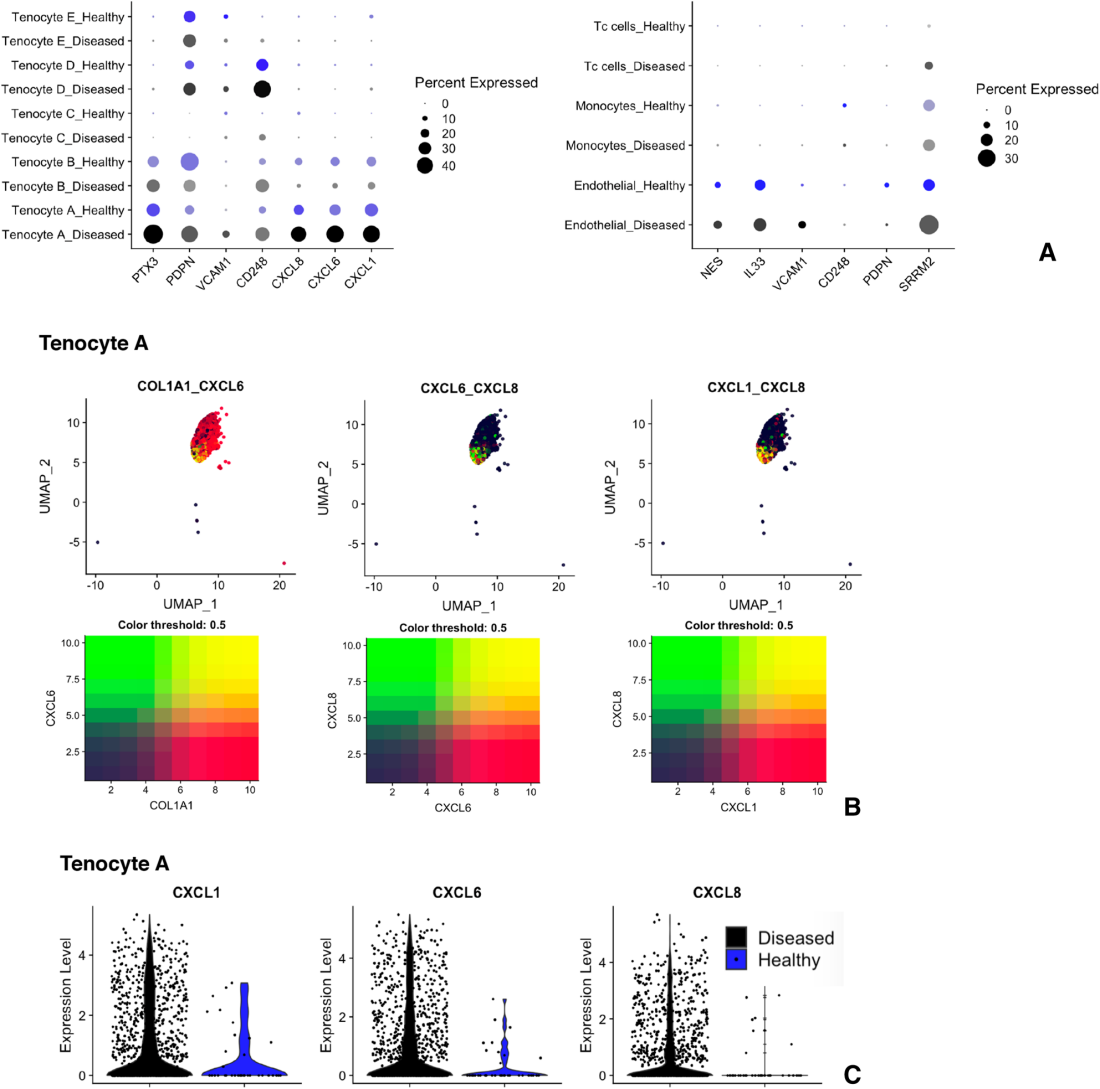
Distinct pro-inflammatory signature of dynamic stromal population in diseased tendon. (**A**) Split dot plot of inflammatory gene expression by matrix associated cells (left) and endothelial cells (right) from diseased (black) and healthy (blue) tendon. The size of circle indicates percentage of cells expressing the gene and the colour intensity indicates the average expression level. (**B**) Combined feature plot of Tenocyte A cluster alone of healthy and diseased cells demonstrating co-expression of CXCL6, CXCL1, and CXCL8 by a subset of cells. (**C**) Split violin plot demonstrating the majority of CXCL6, CXCL1, and CXCL8 expressing cells are from diseased tendon.

The average gene expression analysis of diseased endothelium (Fig. 5) revealed increased expression of *ICAM1* and the chemoattractants *CXCL12, CCL2, SELE* (Selectin-E). When the expression of selected alarmin genes was analysed, *SRRM2, VCAM1, IL33*, and *NES* were found to be increased in diseased versus healthy endothelium (Fig. 6A). Diseased Tenocyte A cells also demonstrated increased expression of pro-inflammatory genes including CXCL1, CXCL6, CXCL8 (Fig. 5) and alarmin genes *CD248*, *VCAM1*, and *PDPN* (Fig. 6A). Co-localisation plots of only the Tenocyte A cluster, shown in Fig. 6B, illustrate that within the Tenocyte A cluster the same cells that expressed *CXCL1* co-expressed *CXCL6* and *CXCL8*. This band of cells predominantly came from diseased tendon samples and very few were found to originate from healthy tendon (Fig. 6C). *PTX3* was up-regulated by diseased cells found predominantly in Tenocyte A, as well as Tenocyte B, Tenocyte C, and Tenocyte D clusters.

Monocytes from diseased tendon were found to have increased expression of chemokine genes CXCL2, CCL2, CCL3, and CCL4 (Fig. 5). Further principal component analysis and dimensionality reduction of this cluster in isolation demonstrated that it was composed of mostly M2 type macrophages and a minority of dendritic cells (Supplemental Figure 4A–C). The macrophages from diseased tendon up-regulated CCL2, CCL3, CCL4, CXCL3, and CXCL8 (Supplemental Figure 4D). Diseased dendritic cells had increased expression of LYZ (lysozyme) and complement genes C1QA, C1QC.

## Discussion

In the absence of valid models of chronic tendon disease, understanding those pathways responsible for tendinopathy has been hampered by conventional methods to isolate crucial cells of interest. In this study, we have combined unbiased single cell gene analysis with surface proteomics^20^ of ex vivo human tendon and described five subpopulations of *COL1A1/2* expressing human tendon cell. To our knowledge this is the first time this approach has been applied to healthy and diseased human tendon.

Five cell clusters were found to express collagen matrix genes. These clusters were initially generated using unbiased analysis of differential gene expression across the integrated data set. The inherent variability in gene expression resulting from technical differences across batches, between different donor individuals and different anatomical sites remains a major limitation of this study. It is possible that these clusters were generated as a result of variation in gene expression that is not directly related to tendon function and as such they represent an artefact of single cell RNA sequencing. In order to start exploring functional differences, the differential expression of tendon matrix genes was compared across the clusters. Hakimi et al. studied the extracellular proteome of human tendon by comparing healthy and torn shoulder (supraspinatus) tissue^19^. As well as identifying a group of proteins up-regulated in diseased tissue, their work provides a catalogue of the most abundant matrix proteins in human tendon. When applied to our data set, the differential expression of these top fifty-six matrix genes further distinguished the Tenocyte A-E clusters (Fig. 2A).

We next looked at differences in cell surface proteomics between the clusters. One of the advantages of CITE-Seq is that it uses oligo-nucleotide conjugated monoclonal antibodies to recognise cell surface proteins at the single cell level. Based on surface expression of an initial panel of stromal markers, we were again able to distinguish between the five clusters, for example Tenocyte A cells were found to be CD10+CD26+CD54+, whereas Tenocyte C were CD146+CD90+ and Tenocyte E cells were CD26+CD10-CD54^low^ (Fig. 3 and Table 2). There is capacity for future work to expand this limited panel of surface markers and investigate whether discrete populations of sorted cells have stable phenotypic differences in vitro. Finally, examples of each of the Tenocytes A–E cluster were evident on immuno-histochemsitry using antibodies that recognise the products of selected genes up-regulated in each cluster (Fig. 5).

Previous single cell transcriptomic studies of mouse^14–16^, and most recently human^21^, muscle have demonstrated a number of different cell matrix associated cell populations including fibro–adipogenic progenitors (FAPs), muscle satellite cells (MuSCs), *ITGA7*+*VCAM1-*/smooth muscle-mesenchymal cells (SMMCs), and scleraxis (*SCX*) expressing cells. Giordani et al. identified up to 8 different cell clusters found in mouse muscle^16^. One of these groups was designated FAPs based on expression of *LY6A, LY6E, PDGFRA*, and *DCN*. Increased expression of *LY6E, PDGFRA* and *DCN*, but not *LY6A*, was found in cells in both Tenocyte D and Tenocyte E from both diseased and healthy human tendon cells (Figs. 1, 2) raising the possibility that these are also types of FAPs. In comparison to Tenocyte E, cells in Tenocyte D exhibited greater expression of tendon collagen genes *COL1A1/2, COL3A1;* microfibril genes *FBN1* and *MFAP5*; and chemo-attractants *CXCL2, CXCL14.* Whereas, Tenocyte E cells expressed *TPPP3* and *PRG4* found in putative tendon stem cells that reside in the paratenon of mice patella tendon and respond to acute tendon injury by producing reparative matrix^17^. In our cohort, these cells co-expressed genes associated with cartilage formation including *ASPN, COMP, PCOLCE2, FMOD, CILP, FN1*, and *PRELP* and were largely found in clusters around disorganised matrix (Fig. 4H). Ongoing study is required to explore whether these differences represent a genuine phenotypic deviation in which reparative *PRG4*+ cells switch to produce a more cartilaginous matrix in the chronic setting; perhaps as a last ditch effort to maintain some structural integrity.

Smooth Muscle-Mesenchymal Cells (SMMCs) were defined in mouse muscle by Giordani et al. as a group of *VCAM1*- cells that express *ITGA7, RGS5*, and *MYL9*^16^. When mouse SMMCs were isolated, a myogenic subset were found to promote muscle growth, forming chimeric myotubes in culture with muscle satellite cells (MuSCs). We found a similar cluster of cells in both healthy and disease human tendon (Figs. 1, 2) and theses tended to be situated around vessels (Fig. 4C). To our knowledge this is the first demonstration of these cells in human tendon and, in keeping with their original description, they do not express endothelial markers found in pericytes^22^. Proteomic analysis revealed surface expression of CD90 and CD146 on these human cells (Fig. 3).

One explanation for the presence of SMMCs may be that tendon samples were taken close the myo-tendinous junction and so muscle tissue was inadvertently included. While this may be a possibility for the healthy hamstring tendon samples, even despite careful dissection distal to the muscle belly, the SMMCs cluster consisted of cells from all tendon samples including the majority taken well distal to the muscle insertion such as digital (toe) extensor tendons. The tendon-bone enthesis interface was also avoided and this is supported by the low expression of *COL2A1* genes across all the clusters (Fig. 2A). Interestingly, *SCX* positive ‘tendon fibroblasts’, similar to Tenocyte B and Tenocyte A, were found in mouse muscle devoid of tendon tissue by Giordani et al. Further work could investigate whether these two seemingly out of place cells are the remnants of respective populations that once had a developmental role or if they still have an adult function, for example a co-ordinated structural response to injury of both muscle and tendon.

One previous study of human tendon used Fluidigm single cell analysis to identify a group of nestin+ putative tendon stem/progenitor cells^23^. This was limited to 71 human tendon cells, all cultured to passage 1 and analysed using 46 gene transcripts. It identified three main cell clusters including a minority expressing increased levels of *NES/CD31/CD146*. In our data set of over 6000 cells obtained immediately ex vivo from human tendon, *NES* (nestin) expression was predominantly found in endothelial cells co-expressing *PECAM-*1, *CD34*, and surface CD34/CD31 (Figs. 1B, 2A and Supplemental Figure 5). This fits with the published nestin+ immunostaining of human Achilles tendon that coincided with blood vessel endothelium. In comparison to ex-vivo cells, we identified eight clusters of in vitro* cultured cells* from three tendon samples, the smallest of which expressed endothelial markers and contained *NES*+ cells (Supplemental Figure 6). It is possible that by culturing cells to passage 1, collagen producing nestin+ cells were preferentially selected by Yin et al. While subsequent mouse models of tendon development and healing demonstrated a role for nestin+ cells, future investigation is required to demonstrate the importance of these endothelial associated cells in adult human tendon.

Tenocyte A and Tenocyte B both expressed high levels of *COL1A1/2* and microfibril genes including fibrillin 1 (*FBN1*), versican (*VCAN*), decorin (*DCN*), elastin microfibril interfacer 1 (*EMILIN1*) and microfibril-associated glycoprotein 2/microfibril associated protein 5 (*MFAP5*). Together these form microfibril chains that have previously been shown to surround a string of tendon resident cells, linking them to the much denser extracellular collagen matrix^11,24,25^. Histological analysis of human tendon revealed long thin chains of Pentraxin 3 (*PTX3)* positively stained cells that may represent these microfibril associated cells (Fig. 4C). The observation that microfibrils bind growth factors such as TGFβ and BMP has led to the hypothesis that they play a role in allowing the relatively few resident tendon cells sample and respond to changes in the surrounding type I collagen matrix^8,9,11^. It would be interesting to explore how microfibril-cell interactions change in response to acute injury and repair versus chronic tendinopathy.

Despite some differences in gene (Fig. 2B) and surface protein expression (Fig. 3) observed between Tenocyte A and Tenocyte B cells, similarities in their matrix gene expression profile (Fig. 2A) questions whether they are two separate populations of tendon cells. It is possible that they share a common progenitor and have developed to perform overlapping roles in response to tissue damage. For example, the Tenocyte A cluster is predominantly composed of diseased tendon cells that co-express chemokine genes *CXCL1, CXCL6*, and the cytokine *CXCL8* (IL-8) and it may be that these cells respond to matrix disruption by recruiting and activating other *COL1A1/2*+ cells.

Alternatively, the two clusters could be explained by variation across samples from male versus female tendon, and/or different anatomical sites. The database comprises the largest number of human tendon cells, healthy tendon cells, diseased tendon cells, and variety of tendon types so far published. While Tenocytes A–E are evident across the data set irrespective of tendon donor, there is variation in the proportion of the five clusters. The contribution of Tenocytes A–E is similar in healthy hamstring tendon, but a greater percentage of Tenocyte A is seen in female (Supplemental Figure 3B) and digital/toe extensor tendon (Supplemental Figure 3C) than Tenocyte E which dominates tibialis posterior and peroneals tendons (foot invertors/evertors respectively). The data set is therefore useful in identifying putative subtypes of human tendon cell but we would caution any conclusions based on the proportions of these subtypes. We are concerned that the tendon processing required for single cell RNA-seq may preferentially select some subtypes while others may fail to survive entirely. Rigorous analysis of how cell proportions vary under different conditions is likely to involve combined approaches that analyse intact tissue immediately ex vivo.

In order to highlight the differences between diseased and healthy tissue, samples from three anatomical sites (digital extensor, peroneal and Achilles tendons) were obtained from patients with clinically end stage tendon disease defined as intractable pain despite all previous non-operative measures. In addition to *CXCL1, CXCL6, and CXCL8*, diseased tendon cells in Tenocyte A demonstrate increased expression of inflammatory/alarmin genes *PDPN* (podoplanin), *VCAM1* (CD106), and *CD248* (Fig. 6). These three were found to be up-regulated in Achilles tendinopathic tissue compared to healthy control tendon^26^. This suggests that in the setting of chronic damage, tendon resident *COL1A1/2*+ cells adopt a pro-inflammatory phenotype. A similar response is seen in endothelial cells from diseased tendon with increased expression of *ICAM1, CXCL12, CCL2, SELE* (Selectin-E), and alarmin genes *SRRM2, VCAM1, IL33*, and *NES* (Fig. 6A). In keeping with previous studies, the predominant immune cell subtypes in tendon are monocytes and Tc cells^26,27^. Chemokine genes *CCL2, CCL3, CCL4, CXCL3*, and *CXCL8* were up-regulated in diseased tendon M2-type macrophages and dendritic cells had increased expression of lysozyme and complement genes.

IL-33 expression has been described in early tendinopathy^28^ while immunostaining of more chronically diseased human Achilles samples found reduced levels of IL-33 compared to healthy or treated patient samples. This was associated with CD68+ macrophages on immuno-flourescence^29^. In our study, *IL33* expression was predominantly found in clusters containing cells with endothelial gene markers and high surface CD31/CD34, with little expression in *CD68*+ clusters or cells expressing collagen genes (Fig. 6A).

*PTX3* was also found to be increased in diseased cells compared to healthy tendon cells in the Tenocyte A cluster (Fig. 6). It encodes a member of the pentraxin protein family and is induced by inflammatory cytokines (e.g. IL-1). *PTX3* has previously been found in endothelial cells and mononuclear phagocytes. In relation to tendon disease, one study found increased *PTX3* in a rat model of tendon responses to mechanical stress^30^ but there is little prior data on its expression in human tendon disease. In order to highlight the observed differences between healthy and diseased tendon, the diseased samples were restricted to extreme cases in which there was end stage tendon disease that remained symptomatic despite multiple founds of non operative therapies. Our observations therefore may not be relevant to earlier stages of disease. Furthermore diseased samples were obtained from a significantly older patient group (Table 1). Age related changes in tendon cell gene expression have been found on bulk sequencing^31^ and it is possible that this explains the observed differential gene expression between healthy and diseased cells. A more thorough analysis of intact tissue is required to appreciate the relevance of these putative mediators in tendon pathophysiology.

## Conclusion

Comparative integrated analysis of the transcriptome of human tendon cells obtained ex vivo from healthy and diseased samples revealed multiple subpopulations expressing matrix associated genes. These included FAP type cells, clusters co-expressing *SCX* and microfibril associated genes, and the first demonstration of *ITGA7*+ smooth muscle-mesenchymal cells (SMMCs) and *TPPP3/PRG4*+ cells in adult human tendon. CITE-Seq surface proteomics identified surface markers by which these cells could be isolated in future. Microfibril associated tenocytes from diseased tendon up-regulated pro-inflammatory markers *CXCL1, CXCL6, CXCL8,* and *PDPN.* Diseased endothelium had increased expression of chemokine and alarmin genes including *IL33* and *PDPN*.

Ongoing work aimed at identifying more specific surface markers by which these cells can be isolated will allow further study of their lineage and phenotypic differences. A combination of approaches that analyse intact tissue ex vivo may prove beneficial in identify key subtypes and signalling pathways involved in different stages of tendon repair and disease.

## Materials and methods

### Collection of tendon samples donated by patients

Tendon biopsies were collected from patients with informed donor consent, in accordance with the Declaration of Helsinki, under ethics from the Oxford Musculoskeletal Biobank (09/H0606/11) and in compliance with National and Institutional ethical requirements. Only waste tissue that would otherwise have been disposed of was collected. Healthy tendon samples were obtained form patients undergoing tendon transfer procedures. These included patients undergoing hamstring (gracilis and semimembranosus) tendon reconstruction of knee anterior cruciate ligament; reconstruction of irreparable Achilles tendon rupture using healthy flexor hallucis longus tendon; and tibialis posterior tendon transfer procedures to improve foot biomechanics. Diseased tendon samples were obtained from patients undergoing surgical exploration and debridement of intractable, chronic painful tendon disease. These included tendinopathic peroneus longus, the Achilles tendon and the extensor digitorum tendon associated with a painful fixed flexion deformity of the proximal inter-phalangeal joint (‘Hammer Toe’).

Tendon samples were obtained distal to the myotendinous junction and proximal to the enthesis. 10 (l) × 10 (w) mm samples were obtained from hamstring, peroneoal, tibialis posterior and Achilles tendons. 5 (l) × 5 (w) mm samples were obtained from digital tendons (toe extensor and flexor hallucis longus).

### Tendon sample digestion

Samples were immediately placed in 4 °C Iscove's Modified Dulbecco's Medium (IMDM) without antibiotics and without FCS. The tendons were rinsed in 1 × PBS, cut axially using a size 10 surgical scalpel into 1 mm^3^ pieces and incubated at 37 °C for 45 min in Liberase (Merck) and 10ul/ml DNAse I (Thermo Scientific). Ham’s F-12 media + 10% FCS was added and the digested tissue passed through a 100 µm cell strainer. The cells suspension was centrifuged at 350 g for 5 min and re-suspended in 1 ml of FCS:DMSO (9:1) freezing medium and immediately placed in -80 °C storage for future batch analysis.

### CITE-seq

Digested healthy and diseased cells were defrosted, washed and re-suspended in 100 µl staining buffer (1 × PBS + 2% BSA + 0.01% Tween). As per the CITE-Seq protocol (https://citeseq.files.wordpress.com/2019/02/cite-seq_and_hashing_protocol_190213.pdf), cells were then incubated for 10 min at 4 °C in human Fc Blocking reagent (FcX, BioLegend). Cells were incubated at 4 °C for a further 30 min with 0.5 µg of TotalSeq-A (Biolegend) monoclonal anti-CD10, anti-CD105, anti-CD146, anti-CD26, anti-CD31, anti-CD34, anti-CD44, anti-CD45, anti-CD54, anti-CD55, anti-CD90 (THY1), anti-CD95, anti-CD73, anti-CD9 and anti-CD140a antibodies (see Supplemental Table 1). In addition, cell suspensions from each sample were incubated with 0.5 µg of the relevant cell hashing antibodies (Biolegend). The cells were then washed three times with staining buffer and re-suspended in 1 × PBS at 1000 cells/µl. The cell suspensions were filtered using a 100 µm sieve. The final concentration, single cellularity and viability of the samples were confirmed using a haemocytometer. Cells were loaded into the chromium controller (10×-Genomics) chip following the standard protocol for the chromium single cell 3’ kit. A combined hashed cell concentration was used to obtain an expected number of captured cells between 5000 and 10,000 cells. All subsequent steps were performed based on the CITE-Seq protocol (https://citeseq.files.wordpress.com/2019/02/cite-seq_and_hashing_protocol_190213.pdf). Libraries were pooled and sequenced across multiple Illumina HiSeq 4000 lanes to obtain a read depth of approximately 30,000 reads per cell for gene expression libraries.

The raw single cell sequencing data was mapped and quantified with the 10 × Genomics Inc. software package CellRanger (v2.1) and the GRCh38 reference genome. Using the table of unique molecular identifiers produced by Cell Ranger, we identified droplets that contained cells using the call of functional droplets generated by cell ranger. After cell containing droplets were identified, gene expression matrices were first filtered to remove cells with > 5% mitochondrial genes, < 200 or > 5000 genes, and > 25,000 UMI. Downstream analysis of Cellranger matrices was carried out using R (3.6.0) and the Seurat package (v 3.0.2, satijalab.org/seurat).

In total, 11,970 cells with an average of 1,700 genes per cell were selected for ongoing analysis. Of these single cells, 6,433 were obtained immediately ex-vivo from digested healthy and diseased tendon. The remainder were cells from P1 culture of two healthy and one diseased tendon to allow comparison with ex-vivo single cell sequencing (Table 1 and Supplemental Table 2, see “Discussion”).

After quality control filtering, data were normalised using SCTransform function for RNA gene expression, hashed antibody (HTO) and surface antibody (ADT) expression level. Based on the HTO expression level, we were able to subsample cells from a particular donor tendon (Supplemental Figure 1). Normalised data from all healthy tendon cells were combined into one object and integrated with data from cells of diseased tendon. Variable genes were discovered using the SCtransform function with default parameters. The FindIntegrationAnchors function command used default parameters (dims = 1:30) to discover integration anchors across all samples. The IntegrateData function was run on the anchorset with default additional arguments. ScaleData and RunPCA were then performed on the integrated assay to compute 15 principal components (PC). Uniform Manifold Approximation and Projection (UMAP) dimensionality reduction was carried out and Shared Nearest Neighbour (SNN) graph constructed using dimensions 1:15 as input features and default PCA reduction^18^. Clustering was performed on the Integrated assay at a resolution of 0.5 with otherwise default parameters which yielded a total of 12 clusters, each composed of cells originating from both healthy and diseased samples across the study patients^32^.

Seurat FindAllMarkers was used to identify positive and negative markers of a single cluster compared to all other cells. Following expression level normalisation using SCTransform function, the average expression level of a feature (e.g. gene) was calculated across each cluster. The minimum percentage of cells in which the feature is detected in the each group was set to 25%. The average log2 fold change threshold was set to at least 1.0 such that the average expression level of a feature would have to be at least double in one cluster compared to another to reach threshold. Significance was determined using Wilcoxon rank sum test with *p *values adjusted based on bonferroni correction applying all features in the data set (*p*_val_adj < 0.05).

### Histology

Immunohistochemistry was performed on a Leica Bond system using the standard protocol F30. The sections were pre-treated using heat mediated antigen retrieval with citrate-based buffer (pH 6, epitope retrieval solution 1) or EDTA based buffer (pH 9, epitope retrieval solution 2) for 20 min. The sections were then incubated with antibody for 30 min at room temperature and detected using an HRP conjugated polymer system in which DAB was used as the chromogen. The sections were counter-stained with haematoxylin and mounted with Aquatex. The following antibodies were used; anti-Pentraxin 3/PTX3 1/500 [MNB1] (Abcam 90806), anti-Cytokeratin 7 1/400 [RCK105] (Abcam 9021), anti-ITGA7 1/500 (Abcam 203254), anti-MGP 1/500 (Abcam 86233), anti-TEM1 1/400 (Abcam 67273), anti-CD31 1/800 [JC70A] (DAKO 20057487), anti-CD68 1/400 (DAKO 20058607), anti-Smooth muscle actin 1/1000 (Abcam 5694), anti-periostin 1/1500 [EPR20806] (Abcam 227049), anti-APOD 1/500 (orb155698), anti-CXCL14 1/1200 (Abcam 137541), anti-Podoplanin 1/750 [D2-40] GTX31231 (lot 821903108).

### Ethical approval

This study received ethical approval from the Oxford Musculoskeletal Biobank (OMB); a research tissue bank with overarching ethical approval from NHS Research Ethics Committee-South Central- Oxford C (reference 19/SC/0134) to collect, store and release musculoskeletal tissue.

## Supplementary information

Supplementary Figure Legends.

Supplementary Table Legends.

Supplementary Figure 1.

Supplementary Figure 2.

Supplementary Figure 3.

Supplementary Figure 4.

Supplementary Figure 5.

Supplementary Figure 6.

Supplementary Figure 7.

Supplementary Table 1.

Supplementary Table 2.

Supplementary Table 3.

Supplementary Table 4.
