# Supplementary material for: Multi-omic single cell analysis resolves novel stromal cell populations in healthy and diseased human tendon
Source: Sci Rep. 2020 Sep 3;10:13939. doi: 10.1038/s41598-020-70786-5 (PMC7471282; doi:10.1038/s41598-020-70786-5)
Supplement: Supplementary file 1 — Supplementary Figure Legends. [file 41598_2020_70786_MOESM1_ESM.docx]

**Supplemental Figure 1.** Cells from a given tendon sample were incubated with CITE-Seq monoclonal antibodies that bind ubiquitous surface proteins and are conjugated to one of eight oligonucleotide barcodes (hash mAb). Post sequencing, the relative ‘expression level’ of these hash barcode mAb were used to identify all the cells from a particular tendon sample. This meant that cells from up to eight tendon samples could be sequenced in a single lane. (**A**) In this example, Seurat v3 was used to select all cells with high ‘expression level’ of hashing hto_Antibody4 and low ‘expression level’ of hto_Antibody1. (**B**) Subsequent Violin plots of the selected cells showed low ‘expression level’ of the other seven hashing antibodies, so confirming the purity of the selected population. This process was performed for all samples before they were integrated as a combined diseased versus healthy data set.

**Supplemental Figure 2.** Principal component analysis and Elbow plot of the integrated ex vivo diseased and healthy data set following quality control.

**Supplemental Figure 3. (A)** Bar graph demonstrating the number of cells from diseased versus healthy tendons grouped by tendon type. Each stacked segment represents the number of cells from individual male (light grey) or female (dark grey) donors. **(B)** Dimension plot of combined diseased and healthy tendon data set split by male versus female donors. **(C)** The percentage of all cell clusters (top) and Tenocyte A-E clusters (bottom) for each of the donor tendon types.

**Supplemental Figure 4. (A)** Dot plot demonstrating relative expression of inflammatory and matrix associated genes across Monocytes and Tenocytes A-E clusters. **(B, C)** Further dimensionality reduction analysis of Monocytes cluster alone reveals at least 2 subtypes; M2 type macrophages and dendritic cells. **(D)** Scatter plots of average gene expression of M2 and DCs highlighting genes with increased expression in diseased (black) versus healthy (blue) tendon.

**Supplemental Figure 5.** CITE-Seq combined feature plot of ex vivo cells demonstrating co-expression of *NES* gene and surface CD31 on cells within clusters expressing endothelial gene markers.

**Supplemental Figure 6.** Integrated CITE-Seq analysis of three tendon samples (two healthy and one diseased) cultured in vitro until passage 1 following mechanical and enzymatic dissociation. **(A)** UMAP demonstrating eight clusters. **(B)** Heat map of average gene expression across the eight clusters of the same gene set for ex vivo cells (see Figure 2). **(C)** Feature plot of selected canonical markers to help identify clusters. **(D)** Combined feature plot of in vitro cultured cells co-expressing NES and COL1A1.

**Supplemental Figure 7. Isotype control immuno-histochemistry of human tendon**

Healthy hamstring tendon was stained with mouse IgG isotype (for anti-CD31, anti-Cytokeratin 7 mAb), rabbit IgG isotype (for anti-Periostin mAb, anti-ITGA7 mAb, anti-CXCL14 mAb, anti-Matrix-gla protein) and rat IgG isotype (for anti-PTX3 mAb).
