# Supplementary material for: Multi-omic single cell analysis resolves novel stromal cell populations in healthy and diseased human tendon
Source: Sci Rep. 2020 Sep 3;10:13939. doi: 10.1038/s41598-020-70786-5 (PMC7471282; doi:10.1038/s41598-020-70786-5)
Supplement: Supplementary file 2 — Supplementary Table Legends. [file 41598_2020_70786_MOESM2_ESM.docx]

**Supplemental Table 1.** CITE-Seq utilises oligonucleotide barcodes conjugated to monoclonal antibodies against cell surface proteins. Fifteen candidate cell surface proteins were targeted. A further eight hashing antibodies, that recognise ubiquitous surface proteins, were exploited to identify cells from one of eight different tendon samples run on a single lane of sequencing.

**Supplemental Table 2.** Cells were obtained from three main sources; ex vivo healthy human tendon, ex vivo diseased tendon and cells cultures to passage 1 (two healthy and one diseased tendon). A total of 11,970 cells were analysed using Seurat V3 post quality control. The right hand table quantifies the distribution of the integrated ex vivo data set across the clusters.

**Supplemental Table 3.** Table listing the top 10 most discriminating genes per cluster identified by scRNA-seq for clusters Tenocyte A-E.

**Supplemental Table 4.** The average gene expression was compared for diseased and healthy cells for each cluster. The 20 genes with the greatest fold change in expression level for diseased compared to healthy cells (black) and healthy versus diseased cells (blue) are listed in summary.
