# Supplementary figures and images for: Multi-omic single cell analysis resolves novel stromal cell populations in healthy and diseased human tendon
Source: Sci Rep. 2020 Sep 3;10:13939. doi: 10.1038/s41598-020-70786-5 (PMC7471282; doi:10.1038/s41598-020-70786-5)

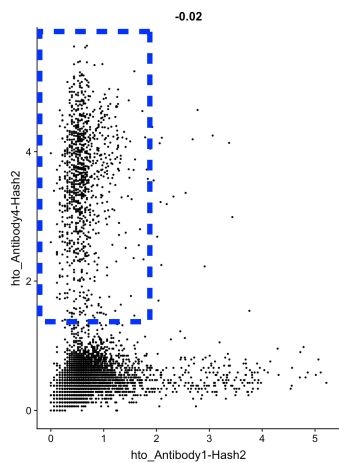

**A**

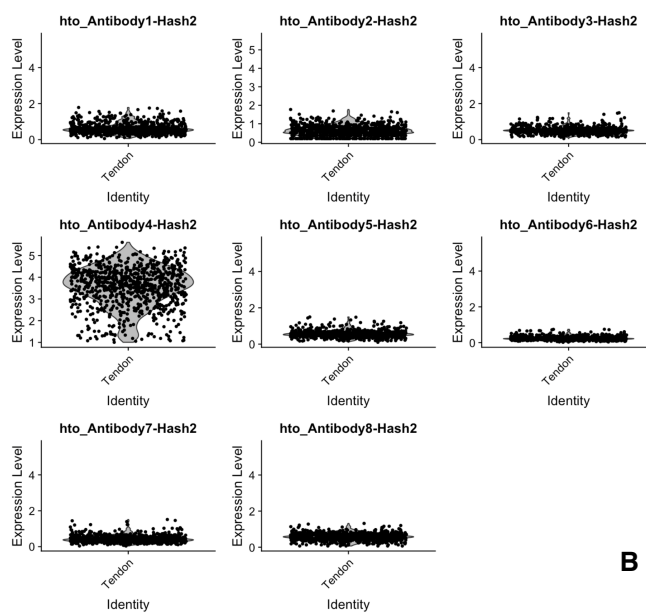

**B**

Supplement: Supplementary file 3 — Supplementary Figure 1. [file 41598_2020_70786_MOESM3_ESM.pdf]

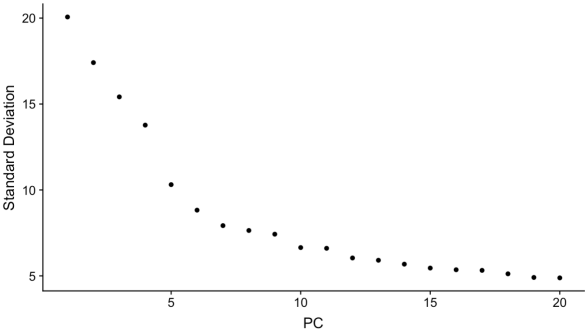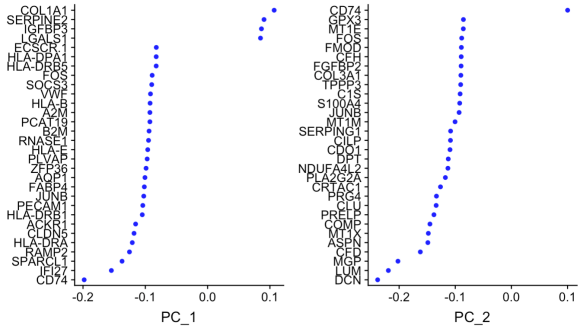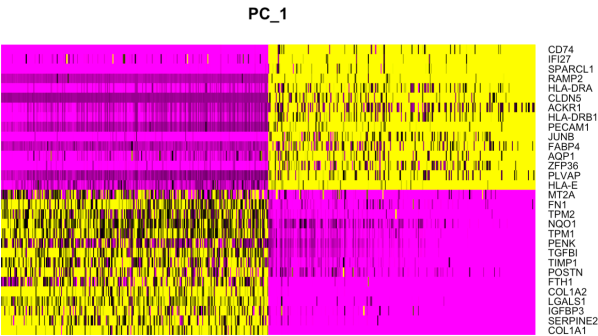

Supplement: Supplementary file 4 — Supplementary Figure 2. [file 41598_2020_70786_MOESM4_ESM.pdf]

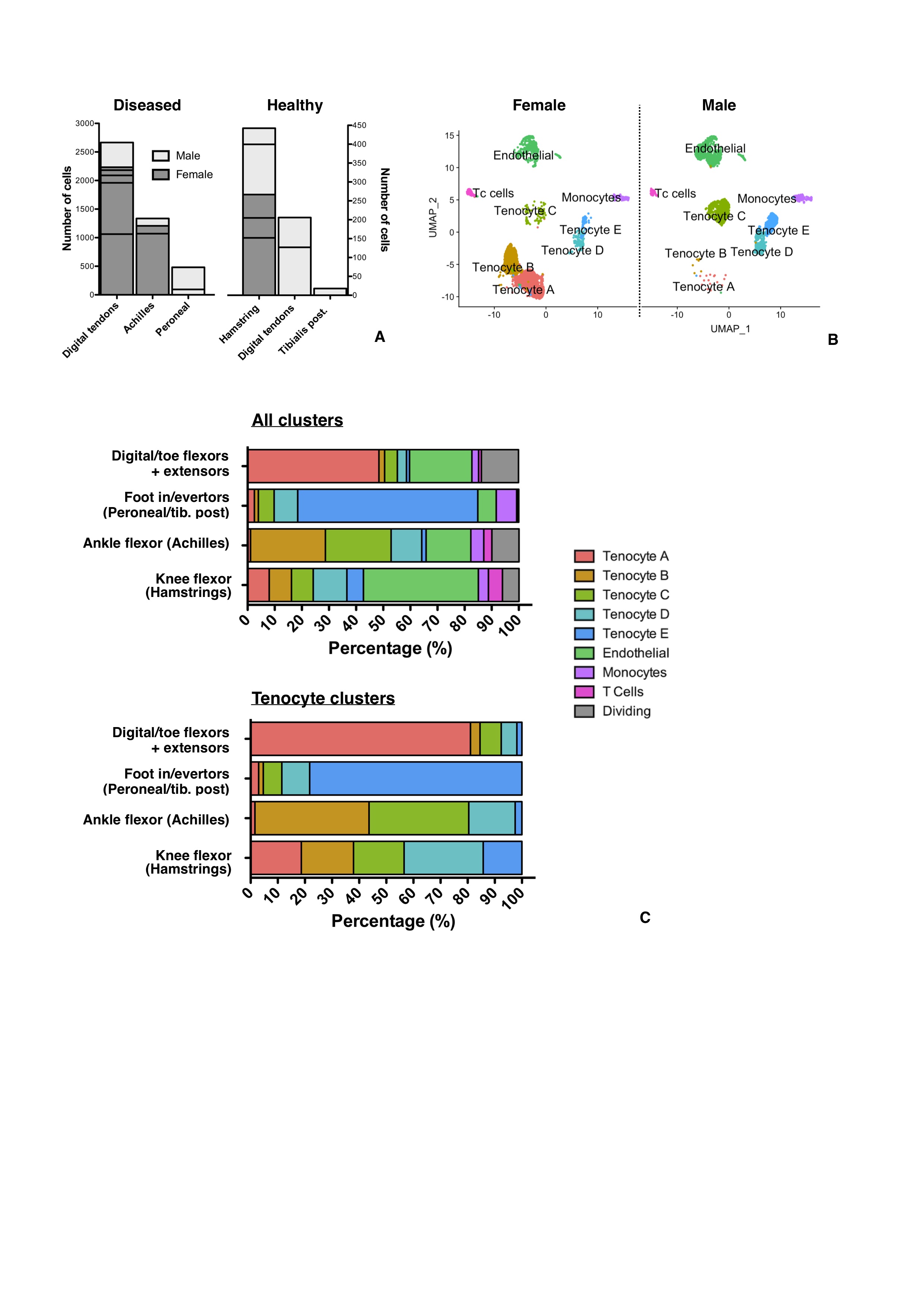

Supplement: Supplementary file 5 — Supplementary Figure 3. [file 41598_2020_70786_MOESM5_ESM.jpg]

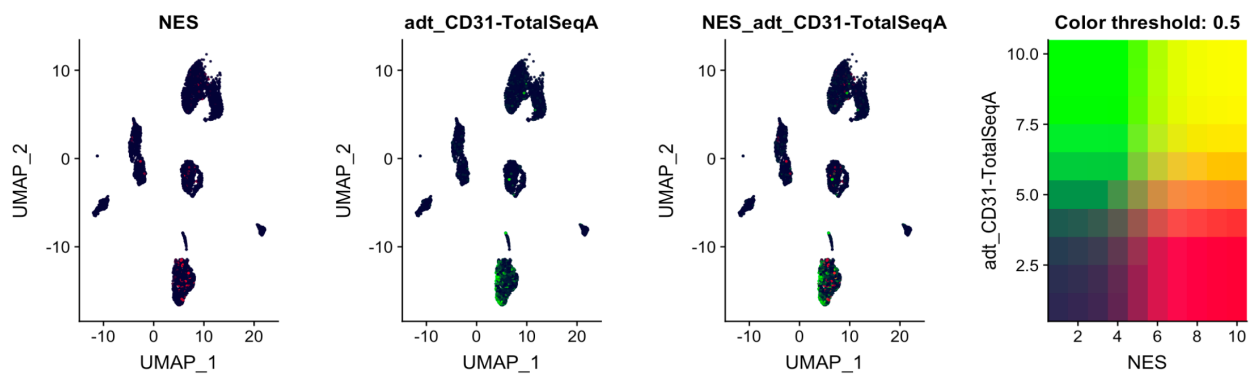

Supplement: Supplementary file 7 — Supplementary Figure 5. [file 41598_2020_70786_MOESM7_ESM.pdf]

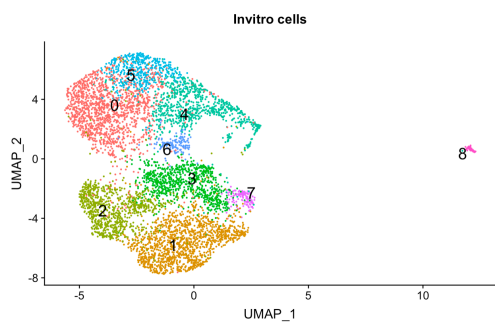

**A**

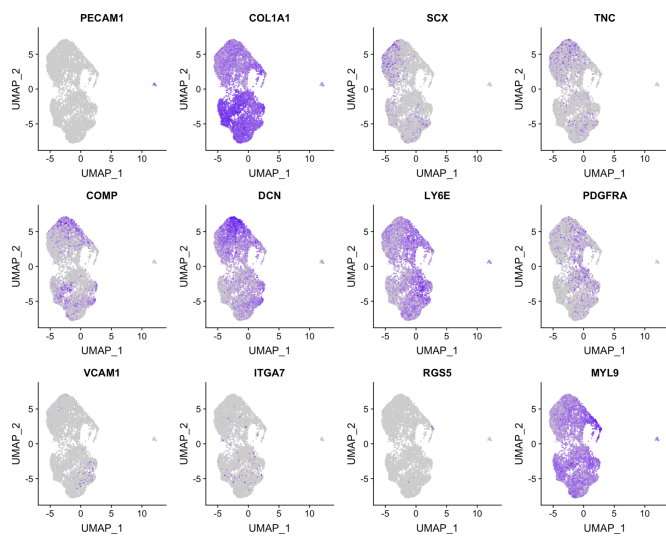

**C**

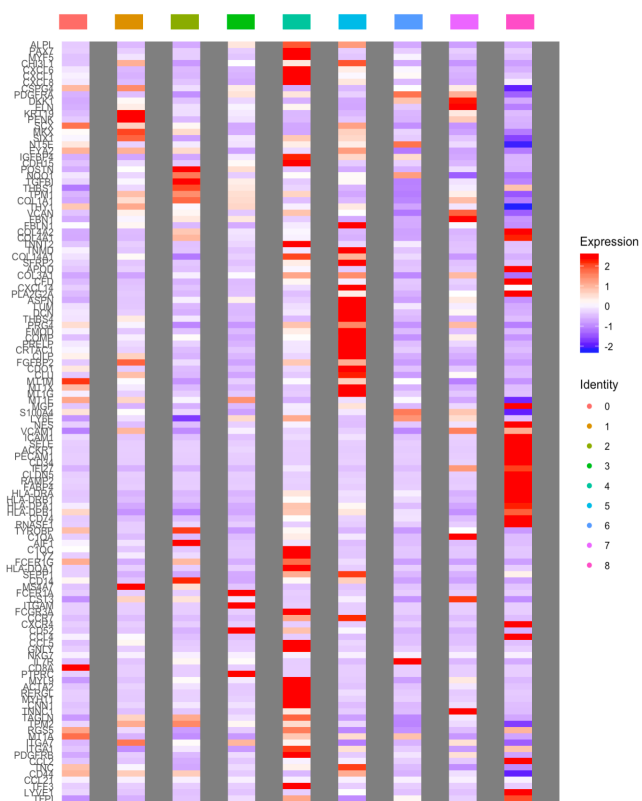

**B**

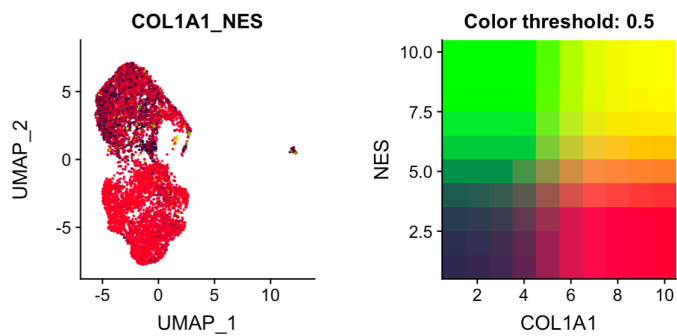

**D**

Supplement: Supplementary file 8 — Supplementary Figure 6. [file 41598_2020_70786_MOESM8_ESM.pdf]

**Mouse IgG**

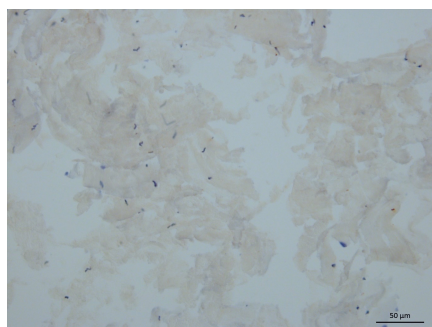

**Rabbit IgG**

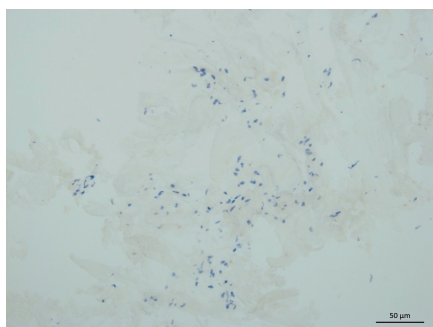

**Rat IgG**

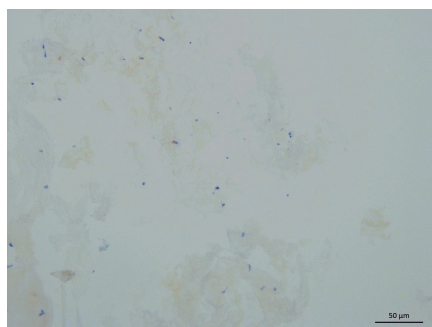

Supplement: Supplementary file 9 — Supplementary Figure 7. [file 41598_2020_70786_MOESM9_ESM.pdf]
