# Supplementary material for: Multi-omic single cell analysis resolves novel stromal cell populations in healthy and diseased human tendon
Source: Sci Rep. 2020 Sep 3;10:13939. doi: 10.1038/s41598-020-70786-5 (PMC7471282; doi:10.1038/s41598-020-70786-5)
Supplement: Supplementary file 10 — Supplementary Table 1. [file 41598_2020_70786_MOESM10_ESM.pdf]

Category	Spec.	Code	Clone	Sequence	Cell hashing	Sequence
TotalSeq™-A	CD10	62	HI10a	CAGCCATTCATTAGG	Hash Antibody 1	GTCAACTCTTTAGCG
TotalSeq™-A	CD105	68	43A3	ATCGTCGAGAGCTAG	Hash Antibody 2	TGATGGCCTATTGGG
TotalSeq™-A	CD146	134	P1H12	CCTTGGATAACATCA	Hash Antibody 3	TTCCGCCTCTCTTTG
TotalSeq™-A	CD26	396	BA5b	GGTGGCTAGATAATG	Hash Antibody 4	AGTAAGTTCAGCGTA
TotalSeq™-A	CD31	124	WM59	ACCTTTATGCCACGG	Hash Antibody 5	AAGTATCGTTTCGCA
TotalSeq™-A	CD34	54	581	GCAGAAATCTCCCTT	Hash Antibody 6	GGTTGCCAGATGTCA
TotalSeq™-A	CD44	125	BJ18	AATCCTTCCGAATGT	Hash Antibody 7	TGTCTTTCCTGCCAG
TotalSeq™-A	CD45	48	2D1	TCCCTTGCGATTTAC	Hash Antibody 8	CTCCTCTGCAATTAC
TotalSeq™-A	CD54	217	HA58	CTGATAGACTTGAGT		
TotalSeq™-A	CD55	383	JS11	GCTCATTACCCATTA		
TotalSeq™-A	CD90 (Thy1)	60	5E+10	GCATTGTACGATTCA		
TotalSeq™-A	CD95 (Fas)	156	DX2	CCAGCTCATTAGAGC		
TotalSeq™-A	CD73	77	TY/ 11.8	CAGTTCCTCAGTTCG		
TotalSeq™-A	CD9	579	HI9a	GAGTCACCAATCTGC		
TotalSeq™-A	CD140a	128	16A1	ATGCGCCGAGAATTA		
