# Supplementary material for: Multi-omic single cell analysis resolves novel stromal cell populations in healthy and diseased human tendon
Source: Sci Rep. 2020 Sep 3;10:13939. doi: 10.1038/s41598-020-70786-5 (PMC7471282; doi:10.1038/s41598-020-70786-5)
Supplement: Supplementary file 11 — Supplementary Table 2. [file 41598_2020_70786_MOESM11_ESM.pdf]

Tendon (11,970)	n	cells
**Healthy**	**8**	**668**
Healthy hamstring	5	
Healthy tibialis posterior	1	
Healthy flexor hallucis longus	2	
**Diseased**	**11**	**5745**
Diseased Achilles	3	
2nd toe extensor tenodon	6	
Diseased peroneus tendon	2	
**Cells (P1 culture)**	3	**5537**
Healthy hamstring (vitro)	1	
Healthy tibialis posterior (vitro)	1	
Diseased toe ext tendon	1	

Cluster	Diseased (%)	Healthy (%)
0 Tenocyte A	22.1	5.7
1 Tenocyte B	14.0	7.3
2 Tenocyte C	12.2	3.7
3 Dividing	10.6	3.7
4 Endothelial	8.1	17.7
5 Tenocyte D	7.7	12.6
6 Tenocyte E	7.0	8.5
7 Endothelial	6.5	9.1
8 Monocytes	4.1	9.4
9 Endothelial	3.5	7.0
10 Tc Cells	1.9	4.0
11 Tenocyte C	1.6	4.8
12 Endothelial	0.7	6.3
