# Supplementary material for: Multi-omic single cell analysis resolves novel stromal cell populations in healthy and diseased human tendon
Source: Sci Rep. 2020 Sep 3;10:13939. doi: 10.1038/s41598-020-70786-5 (PMC7471282; doi:10.1038/s41598-020-70786-5)
Supplement: Supplementary file 12 — Supplementary Table 3. [file 41598_2020_70786_MOESM12_ESM.pdf]

	p_val	avg_logFC	pct.1	pct.2	p_val_adj	cluster	gene
1	<0.001	2.614496074	0.557	0.07	<0.001	Tenocyte A	CHI3L1
2	<0.001	2.189514384	0.419	0.028	<0.001	Tenocyte A	CXCL6
3	<0.001	1.860479414	0.739	0.141	<0.001	Tenocyte A	PENK
4	<0.001	1.501739049	0.983	0.331	<0.001	Tenocyte A	SERPINE2
5	<0.001	1.46218899	0.513	0.065	<0.001	Tenocyte A	KRT19
6	<0.001	1.378995298	0.861	0.217	<0.001	Tenocyte A	HMGA1
7	<0.001	1.230913862	0.598	0.07	<0.001	Tenocyte A	CLDN11
8	<0.001	1.225501712	0.855	0.291	<0.001	Tenocyte A	CTHRC1
9	<0.001	1.966427499	0.418	0.049	<0.001	Tenocyte A	CXCL1
10	<0.001	1.325932778	0.478	0.074	<0.001	Tenocyte A	PTX3
11	<0.001	2.051105215	0.957	0.178	<0.001	Tenocyte B	KRT7
12	<0.001	2.036007488	0.977	0.308	<0.001	Tenocyte B	POSTN
13	<0.001	1.714567079	0.988	0.368	<0.001	Tenocyte B	NQO1
14	<0.001	1.573908632	0.996	0.56	<0.001	Tenocyte B	COL1A1
15	<0.001	1.525675193	0.994	0.426	<0.001	Tenocyte B	TGFBI
16	<0.001	1.470683539	0.994	0.338	<0.001	Tenocyte B	IGFBP3
17	<0.001	1.460785661	1	0.601	<0.001	Tenocyte B	TPM1
18	<0.001	1.383796109	0.855	0.185	<0.001	Tenocyte B	THBS1
19	<0.001	1.272832029	0.953	0.29	<0.001	Tenocyte B	SERPINE1
20	<0.001	1.246807288	0.992	0.525	<0.001	Tenocyte B	CRYAB
21	<0.001	3.470685039	0.926	0.253	<0.001	Tenocyte C	ACTA2
22	<0.001	3.083947381	0.719	0.036	<0.001	Tenocyte C	RGS5
23	<0.001	2.039161907	0.961	0.645	<0.001	Tenocyte C	MYL9
24	<0.001	1.985923841	0.69	0.023	<0.001	Tenocyte C	LGI4
25	<0.001	1.974693468	0.574	0.013	<0.001	Tenocyte C	MYH11
26	<0.001	1.952257961	0.457	0.011	<0.001	Tenocyte C	ACTG2
27	<0.001	1.860981646	0.665	0.043	<0.001	Tenocyte C	PPP1R14A
28	<0.001	1.829557964	0.987	0.668	<0.001	Tenocyte C	TAGLN
29	<0.001	1.951969391	0.549	0.1	<0.001	Tenocyte C	MT1A
30	<0.001	1.954569143	0.702	0.202	<0.001	Tenocyte C	IGFBP5
41	<0.001	3.048672267	0.865	0.21	<0.001	Tenocyte D	CFD
42	<0.001	3.048252827	0.51	0.04	<0.001	Tenocyte D	CXCL14
43	<0.001	1.950916232	0.791	0.131	<0.001	Tenocyte D	CFH
44	<0.001	2.450522506	0.572	0.074	<0.001	Tenocyte D	SFRP2
45	<0.001	2.063552244	0.888	0.352	<0.001	Tenocyte D	C1S
46	<0.001	2.065145483	0.819	0.3	<0.001	Tenocyte D	SERPINF1
47	<0.001	1.938392449	0.538	0.081	<0.001	Tenocyte D	SFRP4
48	<0.001	3.204963656	0.483	0.077	<0.001	Tenocyte D	APOD
49	<0.001	2.582692349	0.529	0.098	<0.001	Tenocyte D	PLA2G2A
50	<0.001	2.100055883	0.837	0.464	<0.001	Tenocyte D	COL3A1
51	<0.001	3.476914673	0.872	0.158	<0.001	Tenocyte E	PRG4
52	<0.001	2.924277642	0.907	0.152	<0.001	Tenocyte E	PRELP
53	<0.001	2.868297988	0.887	0.059	<0.001	Tenocyte E	CRTAC1
54	<0.001	2.764885728	0.791	0.042	<0.001	Tenocyte E	CILP
55	<0.001	2.551671438	0.65	0.021	<0.001	Tenocyte E	FGFBP2
56	<0.001	2.941513069	0.92	0.339	<0.001	Tenocyte E	COMP
57	<0.001	2.526675663	0.985	0.437	<0.001	Tenocyte E	LUM
58	<0.001	3.453029763	0.965	0.604	<0.001	Tenocyte E	MT1X
59	<0.001	3.288676789	0.974	0.579	<0.001	Tenocyte E	CLU
60	<0.001	2.690880834	0.448	0.057	<0.001	Tenocyte E	MT1G
