# Supplementary material for: Multi-omic single cell analysis resolves novel stromal cell populations in healthy and diseased human tendon
Source: Sci Rep. 2020 Sep 3;10:13939. doi: 10.1038/s41598-020-70786-5 (PMC7471282; doi:10.1038/s41598-020-70786-5)
Supplement: Supplementary file 6 — Supplementary Figure 4. [file 41598_2020_70786_MOESM6_ESM.pdf]

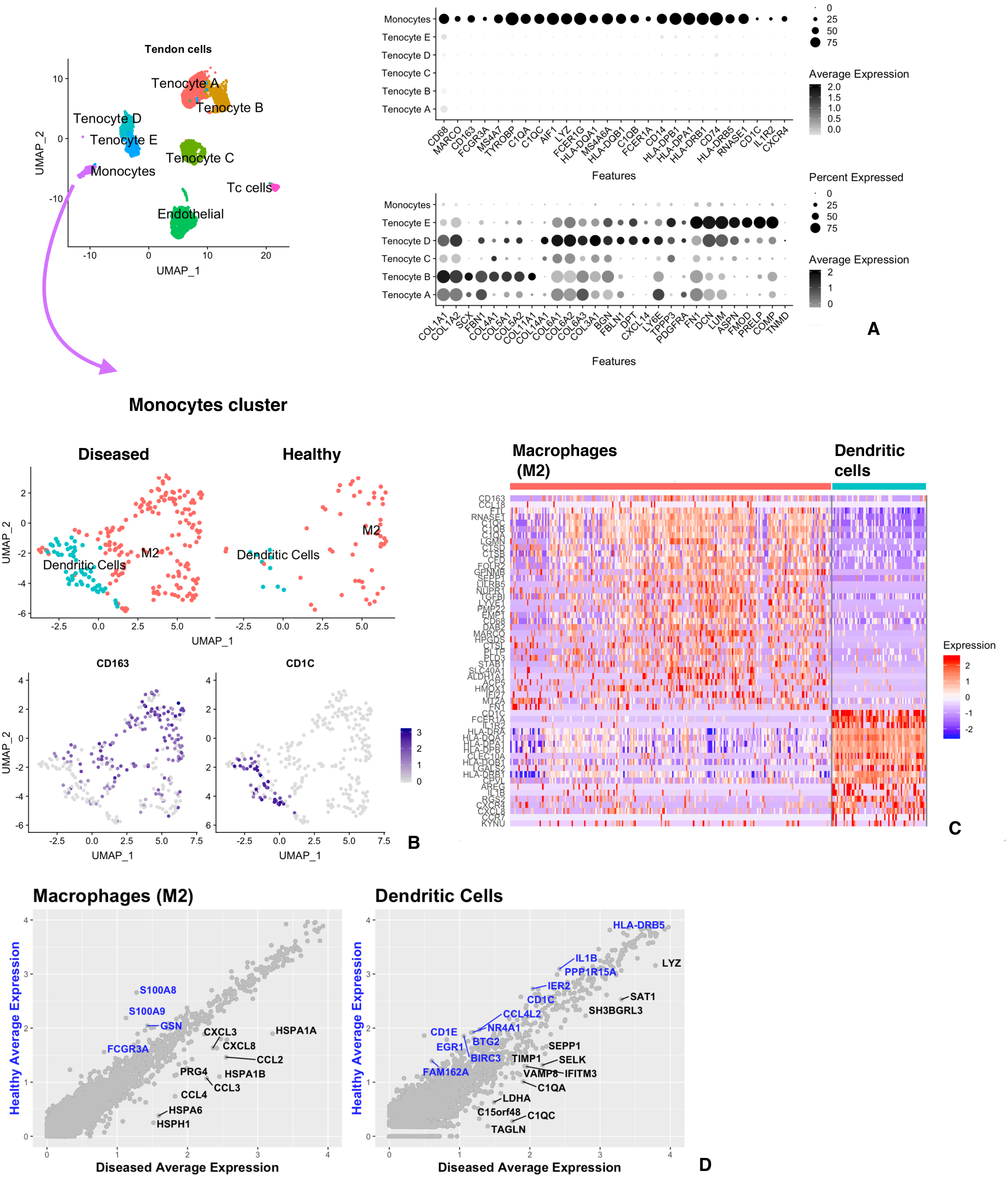
Tendon cells
Tenocyte A
Tenocyte B
Tenocyte C
Tenocyte D
Tenocyte E
Monocytes
Tc cells
Endothelial
UMAP_1
UMAP_2
Monocytes
Tenocyte E
Tenocyte D
Tenocyte C
Tenocyte B
Tenocyte A
CD68 MARCO CD163 FCGR3A MS4A7 TYROBP C1QA C1QC AIF1 LYZ FCER1G HLA-DQA1 MS4A6A HLA-DQB1 C1QB FCER1A CD14 HLA-DPB1 HLA-DPA1 HLA-DRB1 CD74 HLA-DRB5 RNASE1 CD1C IL1R2 CXCR4
Features
Percent Expressed
0
25
50
75
Average Expression
2.0
1.5
1.0
0.5
0.0
COL1A1 COL1A2 SCX FBN1 COL4A1 COL5A1 COL5A2 COL11A1 COL14A1 COL6A1 COL6A2 COL6A3 COL3A1 BGN FBLN1 DPT CXCL14 LY6E TPPP3 PDGFRA FN1 DCN LUM ASPN FMOD PRELP COMP TNMD
Features
Percent Expressed
0
25
50
75
Average Expression
2
1
0
A
Monocytes cluster
Diseased
Healthy
Dendritic Cells
M2
UMAP_1
UMAP_2
CD163
CD1C
UMAP_1
UMAP_2
B
Macrophages (M2)
Dendritic cells
Expression
2
1
0
-1
-2
C
Macrophages (M2)
S100A8
S100A9
GSN
FCGR3A
CXCL3
CXCL8
HSPA1A
CCL2
PRG4
HSPA1B
CCL3
CCL4
HSPA6
HSPH1
Healthy Average Expression
Diseased Average Expression
Dendritic Cells
HLA-DRB5
IL1B
PPP1R15A
LYZ
IER2
CD1C
SAT1
SH3BGRL3
CCL4L2
NR4A1
CD1E
BTG2
EGR1
BIRC3
SEPP1
TIMP1
SELK
FAM162A
VAMP8
IFITM3
C1QA
LDHA
C15orf48
C1QC
TAGLN
Healthy Average Expression
Diseased Average Expression
D
